# Internet use and attitude toward aging among Chinese older adults: the mediating role of health

**DOI:** 10.3389/fpubh.2024.1442075

**Published:** 2025-01-15

**Authors:** Xuehui Wang

**Affiliations:** School of Social Development and Public Policy, Fudan University, Shanghai, China

**Keywords:** older adults, Internet use, attitudes toward aging, self-rated health, physical health, mental health

## Abstract

**Objectives:**

This research seeks to explore the relationship between Internet use and attitudes toward aging among older adults in China, with a particular emphasis on the mediating role of health in this correlation.

**Methods:**

A national survey of 10,858 Chinese adults aged 60 and above was conducted, employing multiple linear regression and structural equation modeling to analyze the impact of Internet use on aging attitudes with health as a mediator.

**Results:**

The research found a significant positive association between Internet use and positive aging attitudes (*β* = −1.420, *p* < 0.001). Health indicators—self-rated, physical, and mental health—were identified as key moderators, with healthier adults benefiting more from Internet use.

**Conclusion:**

This research reveals a positive association between Internet use and attitudes toward aging among older adults in China. These findings hold substantial implications for encouraging active and healthy aging in the digital era, underscoring the value of advocating for digital engagement among seniors as a means to improve their quality of life.

## Introduction

The 21st century is marked by a significant demographic shift: the rapid aging of the population and the accelerated progression of digital technologies (1). This dual transformation presents significant challenges for China, home to the largest older adult population globally (2). In response, the Chinese government has made the well-being and quality of life of the older adult a priority, adopting active and healthy aging strategies to counteract the effects of an aging demographic (3). These strategies highlight the importance of social engagement, with the internet and new media being pivotal in promoting digital interaction and social involvement (3).

The World Health Organization (WHO) launched the concept of active aging in 2002, underscoring the significance of optimizing health, social participation, and security to enhance the quality of life throughout the aging process (4). This affirmative approach challenges the negative stereotypes of aging, focusing on its potential for a rewarding and satisfying experience (5). Digital technologies, especially the internet, are essential facilitators of active aging, offering older adults beneficial experiences and outcomes (6). Studies show that internet use can significantly improve well-being in older adults by reducing loneliness and depression and offering platforms for psychological and social enrichment (7, 8).

Attitudes toward aging play a pivotal role in the life quality and well-being of older adults (9). Positive attitudes can slow cognitive decline and enhance behavior, while negative attitudes can lead to perceptions of poor health, social isolation, and depression (10, 11). Turkish research indicates that these attitudes significantly predict the quality of life (QOL) in older adults (10). However, the influence of internet use on aging attitudes within older population remains insufficiently researched, and the dynamics underlying the connection between Internet use and attitudes are largely uncharted.

This study investigates the correlation between internet use and attitudes toward aging among older population in China, using national survey data to evaluate the impact of online engagement on their perceptions of aging. Additionally, it examines if health status mediates this relationship. Attitude toward aging reflect an individual’s personal evaluation of the aging process, with both positive and negative aspects (12). Tools such as Kogan’s Attitude toward Old People Scale (13), Lawton’s Philadelphia Geriatric Center Morale Scale (14), and Laidlaw’s Attitude to Aging Questionnaire (AAQ) (15) are utilized to quantify these attitudes. Factors known to influence these attitudes include physical health, cognitive function, socio-demographics, and education (16–18), with social support being a significant external contributor, where older adults with strong social networks report greater happiness and more positive aging attitudes (19, 20).

The internet’s potential to enhance older adults’ life quality is substantial (21), but it has not been extensively examined in the relationship between internet use and attitude toward aging (22). It provides opportunities for social engagement and positive attitude development towards aging (21). Internet use can help older adults gain knowledge and skills, boosting self-efficacy and fostering a positive view of aging (23, 24). The WHO defines active aging with an emphasis on social participation, achievable through interaction and social roles (25–27). Internet use can strengthen communication and social involvement, improving health and life quality for seniors (28). Self-rated health, physical health, and mental health are key to well-being, with internet use showing benefits for all (29–31). Poorer self-rated health is associated with more negative aging attitudes (29), while better physical health is linked to more optimistic aging perceptions (32, 33). Mental health benefits from internet use, which can reduce loneliness and depression and improve mental well-being (34, 35). Based on these findings, the study proposes several hypotheses:

*H1*: Internet use exerts a substantial influence on shaping the attitudes of older adults towards aging.

*H2*: Internet use has a beneficial impact on the self-assessed health of older adult individuals, and this self-assessed health serves as a mediator in the relationship between Internet use and attitudes toward aging.

*H3*: Internet use positively influences the physical well-being of older adults, and this physical health acts as a mediating factor in the relationship between Internet use and attitudes towards aging.

*H4*: Internet use has a beneficial impact on the mental health of the older adult, and this mental well-being plays a mediating role in the influence of Internet use on the attitudes towards aging that older adults maintain.

## Methods

### Data and study sample

The study’s sample was derived from the 2018 Chinese Longitudinal Aging Social Survey (CLASS), a comprehensive national research initiative conducted by Renmin University of China. Focusing on older adults aged 60 and above in Mainland China, the survey covers 28 provinces. The 2018 CLASS questionnaire included questions on internet access and usage among the older adult, representing the largest and most nationally representative dataset of internet use for older adults in China. The final sample was drawn from 134 counties and districts across 462 communities, yielding 11,418 valid responses with an impressive response rate of 95%.

In this study, missing data primarily concerned the attitudes toward aging variable, with 1,449 missing values in total. Analysis showed these omissions were randomly distributed across regions. To address this, we employed Multiple Imputation (MI), a recognized effective method for handling missing data (36). Using multivariate normal regression for the interpolation analysis, we performed ten imputations, resulting in 10,858 samples post-MI.

### Measures

#### Dependent variable

The dependent variable in this study was the attitude toward aging, measured with the AAQ (Attitude to Aging Questionnaire) (37). The scale comprises eight items rated on a 5-point Likert scale from “strongly disagree” to “strongly agree.” Four items, such as “I would be happy to participate in some of the work of the village/neighborhood committee if the opportunity arises”, assess a positive attitude toward aging, with higher scores indicating a more favorable view. Another four items, like “Society is changing so fast that I’m having a hard time adapting to the changes”, evaluate negative attitudes, also scored to reflect a more favorable attitude when reversed. The AAQ demonstrated good internal consistency and reliability with a Cronbach’s alpha of 0.81. The scale ranges from 8 to 40, where higher scores denote a more positive attitude toward aging.

#### Independent variable

The independent variable in this study was the older adult’s internet use, assessed by asking respondents if they had used the internet at the time of the survey through any device, including smartphones, computers, or tablets. We categorized responses into “online” (assigned a value of “1”) for those who reported using the internet daily, weekly, monthly, or several times a year, and “not online” (value “0”) for those who never used the internet.

#### Mediating variable

Health status of older adults served as a mediating variable in this study. We measured it using multiple indicators, including self-rated health, which is a well-established and robust predictor of health status, functional capacity, and mortality risk among older adults (38). Self-rated health was evaluated by asking, “How do you rate your current physical health?” Responses were categorized as “good health” (assigned a value of “1”) for “very healthy”, “relatively healthy”, and “healthy”, and “poor health” (value “0”) for “relatively unhealthy” and “very unhealthy.”

Physical health was assessed using the IADL scales (Instrumental Activities of Daily Living), which consist of 10 items scored on a 3-point scale. Participants were asked about their difficulty with activities such as telephoning, cleaning, using stairs, walking outside, public transportation, shopping, managing money, lifting 5 kg, cooking, and housekeeping. Responses of “need some help” and “need help” were categorized as “having difficulty” (value “0”), and “no need for help” as “no difficulty” (value “1”). The item scores were summed, with a range of 0–10, where higher scores indicate fewer difficulties in performing IADL. The IADL exhibited strong internal consistency and reliability, with a Cronbach’s alpha coefficient of 0.89.

Depression was assessed using the 9-item CES-D scale revised by Silverstein (39), a key indicator of mental health, especially in older adults where depression is most prevalent (40). The CESD-9 measures the severity of depressive symptoms with nine questions scored from 1 (no) to 3 (often) (41). Two positively worded items are reverse-scored, resulting in a total score of 9 to 27, where higher scores reflect more severe depressive symptoms and poorer mental health. The CES-D showed robust internal consistency, with a Cronbach’s alpha of 0.76.

#### Control variables

We controlled for socio-demographic factors including age, gender (*0 = female, 1 = male*), marital status (*0 = unmarried/divorced/widowed, 1 = married*), education level (*0 = illiterate to 16 = university or higher*), and residence (*0 = rural, 1 = urban*), along with income, number of sons, and number of daughters. Recognizing the link between life satisfaction and attitudes toward aging, we also controlled for life satisfaction. This was measured by the question, “Overall, are you satisfied with your current life?” with responses categorized as “satisfied” (“very satisfied”, “relatively satisfied”, “average”, “value 1”) and “unsatisfied” (“relatively unsatisfied”, “rather unsatisfied”, “value 0”).

### Statistical analysis

In this study, we used Stata 16.0 for statistical analyses. We began with descriptive statistics for all variables. Then, we conducted bivariate correlation analysis to explore the link between internet use and attitudes toward aging. To tackle endogeneity, we applied the two-stage least squares method. Finally, Structural Equation Modeling (SEM) was employed to examine the mediating roles of self-rated health and mental and physical health.

The potential for endogeneity in our regression model is a valid concern, as it is possible that older adults with a positive attitude toward aging may be more inclined to use the Internet, suggesting a reverse causality. To address this and ensure the robustness of our results, we employed the instrumental variable method. This approach necessitates the selection of instruments that are both relevant-meaning they should have a significant correlation with the independent variable-and exogenous-indicating they should not be directly linked to the error term of the model.

In the analysis, we selected “GDP per capita by province in 2018” and “the primary income source of the older adult” (with a binary variable where pension income is coded as 1 and other income sources as 0) as our instrumental variables. These variables were selected because they are likely to influence the propensity of older adult individuals to use the Internet without directly impacting their attitudes toward aging.

## Results

### Descriptive analysis

Table 1 shows the sample characteristics with descriptive statistics, including counts, ranges, means, and frequencies for the variables. The mean attitude toward aging score was 24.11, on a scale from 8 to 40. Only 18.95% of participants reported internet use in 2018. Regarding self-rated health, 83.68% of respondents rated their health as good. The average scores for physical health and mental health were 9.17 (scale: 0–10) and 14.22 (scale: 9–27), respectively.

**Table 1:** Characteristics of the related variables (*N* = 10,858).

Variables		Range	Mean	Percentage
Dependent variable	Attitude toward aging	8–40	24.11	
Independent variable	Internet use	0–1		
	Yes			18.95
	No			81.05
Mediating variables	Self-rated health	0–1		
	Unhealthy			16.32
	Healthy			83.68
	Physical health			
	IADL scores	0–10	9.17	
	Mental health			
	CES-D scores	9–27	14.22	
Control variables	Age	60–108	71.25	
	Gender			
	Female			49.43
	Male			50.57
	Marital status			
	No spouse			30.01
	With a spouse			69.99
	Education level	0–16	5.88	
	Residence			
	Rural			46.56
	Urban			53.44
	Logarithm of individual income	4.25–13.82	8.42	
	Number of sons	0–8	1.37	
	Number of daughters	0–7	1.21	
	Life satisfaction	0–1		
	Dissatisfied			7.68
	Satisfied			92.32

For the control variables, the respondents’ mean age was 71.25 years, with ages ranging from 60 to 108. Nearly half, 49.43%, were female, and 69.99% were married or living with a partner. Most had less than a high school education, averaging 5.88 years of schooling (0 to 16 years). 53.44% resided in urban areas. The mean logarithmic annual income was 8.42 (ranging from 4.25 to 13.82). Respondents had an average of 1.37 sons (0 to 8) and 1.21 daughters (0 to 7). Regarding life satisfaction, a vast majority, 92.32%, were satisfied with their current life.

### Bivariate relationships among key variables

We conducted a bivariate correlation analysis to explore the relationships between internet use, self-rated health, physical and mental health, and attitudes toward aging (see Supplementary Table S1). Key findings include: a significant positive correlation between internet use, self-rated health, and IADL scores with more favorable attitudes toward aging, and a negative correlation with depression scores. Additionally, internet use correlated positively with self-rated health and IADL scores, and negatively with depression scores. Self-rated health also showed a positive link with IADL scores and a negative association with depression scores. Lastly, there was a notable negative correlation between IADL scores and depression scores, highlighting the complex interplay between these variables.

### Impact of Internet use on the attitudes toward aging among older adults

The least-squares method revealed significant correlations between internet use and attitudes toward aging (see Table 2). Higher age (*β* = −0.021, *p* = 0.001) and more sons (*β* = −0.095, *p* = 0.040) were linked to less positive attitudes toward aging. Being married (*β* = 0.268, *p* = 0.005), higher education levels (*β* = −0.053, *p* < 0.001), and higher income (*β* = −0.188, *p* < 0.001) increased the likelihood of positive attitudes. Life satisfaction also predicted positive attitudes toward aging (*β* = −2.784, *p* < 0.001). Gender (*β* = −0.088, *p* = 0.289), residence (*β* = −0.089, *p* = 0.323), and number of daughters (*β* = −0.026, *p* = 0.509) showed no significant association with attitudes toward aging. Crucially, after controlling for demographics, internet use (*β* = 1.420, *p* < 0.001) was found to be positively related to positive attitudes toward aging among China’s older adult.

**Table 2:** Associations of Internet use with attitude toward aging among older adults (*N* = 10,620).

Variables	*β*	SE	t	*p*	95% CI	
Age	−0.021	0.006	−3.34	0.001	−0.034	−0.009
Gender	0.088	0.083	1.06	0.289	−0.074	0.251
Marital status	0.268	0.096	2.80	0.005	0.080	0.455
Education level	0.053	0.011	4.65	<0.001	0.031	0.076
Residence	−0.089	0.090	−0.99	0.323	−0.266	0.088
Logarithm of family income	0.188	0.032	5.88	<0.001	0.125	0.251
Number of sons	−0.095	0.046	−2.05	0.040	−0.186	−0.004
Number of daughters	−0.026	0.040	−0.66	0.509	−0.105	0.052
Life satisfaction	2.784	0.152	18.36	<0.001	2.487	3.082
Internet use	1.420	0.112	12.61	<0.001	1.199	1.640

### Robustness test

To ensure the stability and reliability of the research results, we conducted a robustness test using the instrumental variable method, with the instruments being “GDP per capita by province in 2018” and “the primary income source of the older adult.” Employing these instruments, our goal was to isolate the impact of internet use on attitudes toward aging while controlling for potential confounders. Table 3 displays the results from the instrumental variable regression analysis. We first confirmed the relationship between our instruments and internet use: Panel A shows that higher provincial GDP per capita in 2018 correlates with increased internet usage among the older adult (*β* = −0.027, *p* < 0.001), and Panel B indicates that pension income as the primary source also raises this likelihood (*β* = −0.120, *p* < 0.001), fulfilling the relevance criterion. In the second stage, we assessed the effect of internet use on attitudes toward aging with the instrumental variables. Both panels suggest that internet use is significantly and positively associated with positive aging attitudes, even after considering demographic variables (Panel A: *β* = 8.917, *p* < 0.001; Panel B: *β* = 4.012, *p* < 0.001). These findings affirm that internet use positively influences older adult attitudes toward aging and effectively address endogeneity concerns.

**Table 3:** Instrumental variable estimation.

Variables	A		B	
	Instrumental variables: GDP per capita by province in 2018		Instrumental variables: the main income resource of the older adult	
	First stage	Second stage	First stage	Second stage
GDP per capita by province in 2018	0.027*** (0.001)			
The main income resource of the older adult			0.120*** (0.009)	
Internet use		8.197*** (0.647)		4.012*** (0.841)
Observations	10,858		10,620	

Both Panel A and Panel B control for the demographic characteristics of older adults. In Panel A, the dependent variable in the first stage is Internet use, with the independent variable being the GDP per capita by province in 2018. The dependent variable in the second stage is the attitude toward aging, with Internet use as the independent variable. In Panel B, the structure is similar: the dependent variable in the first stage remains Internet use, but now the independent variable is the primary income source of the older adult. Again, in the second stage, the dependent variable is the attitude toward aging, with Internet use as the independent variable. *p < 0.05, **p < 0.01, ***p < 0.001.

Additionally, this study conducted tests to ensure the instruments were not weak. The first-stage regression’s *F*-value should exceed 10 as an empirical threshold (42). The Weak Instrument Variable (WIV) test *F*-values for Panel A and Panel B were 42.56 and 73.23, respectively, meeting this standard. Therefore, no weak instrumental variables were present. Moreover, a Stock-Yogo weak identification test was used, where an F-threshold of 16.38 at the 10% expected maximum level indicates a weak instrument with one endogenous and one instrumental variable (43). The F-threshold values from this study’s regressions exceeded 40, indicating a minimal chance of a weak instrumental variable issue.

After confirming the validity of the instrumental variables, both a Hausman test and ordinary least-squares regression (OLS) were performed to assess the endogeneity of Internet use (see Table 4). Using “GDP per capita by province in 2018” as an instrument, the Hausman test *p*-value was 0.173, failing to reject the null hypothesis of no difference between OLS and two-stage least-squares (2SLS) estimates. Similarly, with “the main income source of the older adult” as the instrument, the p-value was 0.095, also not rejecting the null. These results suggest no significant endogeneity in the Internet use variable. Therefore, OLS estimation is deemed more efficient than 2SLS for evaluating the impact of Internet use on attitudes toward aging, as it does not introduce systematic bias.

**Table 4:** Weak instrument variable test and Hausman test.

Test		Instrumental variable	
		GDP per capita by province in 2018	The main income resource of the older adult
Weak IV test	*F*	409.99	196.73
Hausman test	*P*	0.173	0.095

### Test and magnitude of the mediating effects of health status

**Figure 1:**
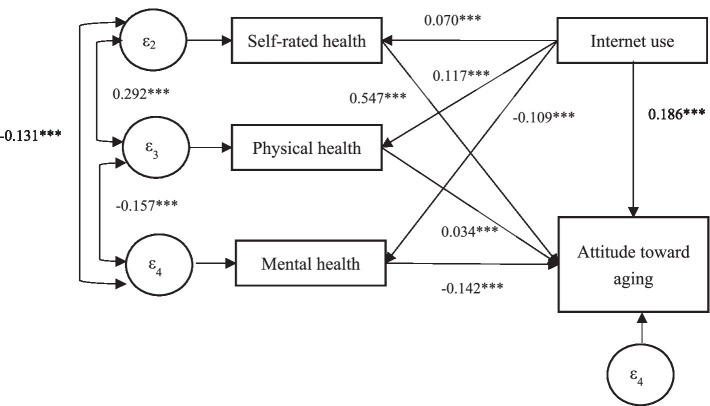
Path analysis of the impact of Internet use on older adult attitudes toward aging. *p < 0.5, **p < 0.01,***p < 0.001; RMSEA < 0.05, SRMR < 0.05, CFI > 0.90, TLI > 0.90.

In this study, we employed Structural Equation Modeling (SEM) to investigate the influence of Internet use on attitudes toward aging among older adults. The SEM analysis, as shown in Figure 1, reveals that Internet use has a significant and positive impact on these attitudes (*β* = 0.186, *p* < 0.001), thereby validating the reliability of our results. Furthermore, our analysis shows that Internet use is positively correlated with enhanced health status, as users report higher levels of self-rated health (*β* = 0.070, *p* < 0.001) and physical health (*β* = 0.117, *p* < 0.001), and lower levels of mental health scores (*β* = −0.109, *p* < 0.001). Additionally, positive attitudes toward aging are linked to increased self-rated health (*β* = 0.547, *p* < 0.001) and physical health (*β* = 0.034, *p* < 0.001), and decreased mental health scores (*β* = −0.142, *p* < 0.001). The SEM results also highlight the reciprocal relationships: a beneficial association between self-rated and physical health (*β* = 0.292, *p* < 0.001), and detrimental correlations between mental health and both physical health (*β* = −0.157, *p* < 0.001) and self-rated health (*β* = −0.131, *p* < 0.001). These insights underscore the intricate interplay between Internet usage, health indicators, and the psychological disposition toward aging.

Table 5 illustrates that health plays a significant mediating role in the relationship between Internet use and attitudes toward aging. The indirect impact of Internet use on attitudes through self-rated health is 0.0408, constituting 6.94% of the total effect. Physical health exerts a more substantial mediating influence, with an indirect effect of 0.0272, or 44.44% of the total effect. Mental health also serves as a mediator, with an indirect effect of 0.0267, representing 23.16% of the total effect. Collectively, these health dimensions emerge as critical mediators, with physical health exhibiting the most pronounced effect. The overall effect of Internet use on fostering positive attitudes toward aging is approximately 0.5337, and the mediating effect through health is 0.0947, accounting for 17.74% of the total effect. These findings underscore the pivotal role of health in shaping older adults’ positive perspectives on aging.

**Table 5:** Decomposition analysis of the impact of Internet use on the attitudes of older adults toward aging.

Independent variable/ Mediating variable	Direct effect	Indirect effect	Total effect	Proportion of indirect effect
Self-rated health	0.547	0.0408	0.5878	6.94%
Physical health	0.034	0.0272	0.0612	44.44%
Mental health	−0.142	0.0267	−0.1153	23.16%
Internet use	0.186	0.0947	0.5337	17.74%

## Discussion

This study explores the relationship between Internet use and attitudes toward aging among China’s older adult population. Our findings, presented in Table 3, confirm the positive influence of Internet use on attitudes toward aging, supporting Hypothesis 1. The Internet’s role in compensating for social relationship losses associated with aging is a key factor. It offers a platform for social support (44), enabling older adults to connect with relatives and friends, thereby alleviating loneliness and reducing fear of aging. The Internet also facilitates the maintenance of existing relationships and the formation of new ones (45, 46), increasing community participation and expanding social networks (47, 48). This helps older adults accumulate social capital and demonstrate their self-worth, enhancing their life perspectives and self-assessment (49).

The mediating effect of self-rated health between Internet use and attitudes toward aging, confirmed by Hypothesis 2, is significant. Internet use among older adults enhances adaptability and self-assurance, improving self-rated health. This aligns with the literature that reduced social activity participation can adversely affect self-rated health (50, 51). Better self-rated health is associated with greater independence and autonomy, fostering a positive outlook on aging.

Hypothesis 3 is also supported by our results, indicating that physical health mediates the relationship between Internet use and attitudes toward aging. The Internet is a valuable tool for accessing health management information, enhancing physical health (30). However, physical limitations can hinder Internet use among older adults (19, 52), suggesting that those in better physical health are more likely to use the Internet and develop a positive attitude toward aging.

Furthermore, our findings support Hypothesis 4, showing that mental health mediates the effect of Internet use on attitudes toward aging. Internet engagement can reduce depression risk and improve mental well-being in older adults (34). The Internet can overcome barriers of time and distance, increasing social interaction frequency and alleviating depression (53, 54).

There is a scarcity of research on the relationship between internet use among the older adult and their attitudes towards aging, with only one existing study exploring the relationship between attitudes towards aging and the acceptance of ICT among older adults (55). The comparison between the study on Western attitudes towards aging and ICT acceptance and the Chinese research on Internet use and aging attitudes reveals cultural and infrastructural disparities. The Western study emphasizes that older adults’ limited tech experience and cautious adoption pose barriers to ICT use for aging in place. In contrast, the Chinese study shows a positive link between Internet use and aging attitudes, suggesting that health improvements from online engagement may drive tech adoption among older adults. Generational differences in ICT acceptance are noted in the West, while in China, a more uniform positive view towards Internet use in aging is observed, possibly due to varying societal values and infrastructures. The widespread internet access in China might contribute to the positive aging attitudes through its impact on health, contrasting with the potential digital divide in Western regions. In essence, these studies underscore how cultural norms and technological reach shape the older adult’s engagement with technology and their perceptions of aging across different global contexts.

## Study limitations

Our study has limitations. The use of 2018 CLASS data provides a snapshot of the influence of Internet use on attitudes toward aging among China’s older adult. While our cross-sectional data does not establish causality, we mitigated endogeneity using the instrumental variable method. There may still be omitted variables due to uncontrollable factors. However, our use of a structural equation model to analyze mediating effects reinforces the significant and positive impact of Internet use on older adults’ attitudes toward aging.

## Conclusion

Our study’s innovative contribution lies in its comprehensive analysis of the mediating effects of self-rated health, physical health, and mental health on the relationship between Internet use and attitudes toward aging. This nuanced understanding can inform targeted interventions to enhance older adults’ Internet literacy and access, improving their attitudes toward aging and overall well-being.

The practical implications of our research are significant. As the older adult population grows, understanding how Internet use can positively influence their attitudes toward aging is crucial. Our findings suggest that promoting Internet use among older adults could improve their health and mental well-being, leading to more optimistic attitudes toward aging. The research indicates that fostering digital engagement among China’s older adult is crucial for promoting active and healthy aging. Policymakers should implement educational programs to familiarize older adults with technology, ensuring these are accessible to diverse age groups. Collaborating with the private sector, they should develop user-friendly technologies that cater to seniors’ needs, enhancing their digital experience. Integrating digital health solutions into healthcare can improve patient outcomes and efficiency. Additionally, supporting online communities for the older adult can combat social isolation. By encouraging Internet usage among seniors, China can enhance their health, well-being, and quality of life, leveraging the digital era to support active aging.

In conclusion, our study provides a unique contribution to the literature by highlighting the importance of Internet use in shaping attitudes toward aging among China’s older adult population. Our findings underscore the need for strategies to encourage Internet use among older adults, with the potential to enhance their health, mental well-being, and overall quality of life.

## Data Availability

The datasets presented in this study can be found in online repositories. The names of the repository/repositories and accession number(s) can be found at: class.ruc.edu.cn.
