# Supplementary material for: Internet use and attitude toward aging among Chinese older adults: the mediating role of health
Source: Front Public Health. 2025 Jan 15;12:1442075. doi: 10.3389/fpubh.2024.1442075 (PMC11774908; doi:10.3389/fpubh.2024.1442075)
Supplement: Supplementary file 1 [file Table_1.DOCX]

Table 1 Correlations between variables (N=10858)

| Variables | Internet use | SRH | IADL | Depression | ATA |
| --- | --- | --- | --- | --- | --- |
| Internet use | 1 |  |  |  |  |
| SRH | 0.0698 | 1 |  |  |  |
| IADL | 0.1165 | 0.2971 | 1 |  |  |
| Depression | -0.1093 | -0.1380 | -0.1677 | 1 |  |
| ATA | 0.2101 | 0.0974 | 0.0956 | -0.1757 | 1 |

*Note.* ATA refers to attitude toward aging. All correlations are statistically significant, p<0.001.
